# Disodium 4,5,6-trihy­droxy­benzene-1,3-disulfonate dihydrate

**DOI:** 10.1107/S1600536810036160

**Published:** 2010-09-15

**Authors:** E. Song, J. Podschun, H. Wilberts, U. Beginn, H. Reuter

**Affiliations:** a Institute of Chemistry, Barbarastrasse 7, D-49069 Osnabrück, Germany

## Abstract

In the title compound, 2Na^+^·C_6_H_4_O_9_S_2_^2−^·2H_2_O, the benzene rings of the 4,5,6-trihy­droxy­benzene-1,3-disulfonate ions, which are stacked parallel to each other forming rods parallel to the *a* axis, are slightly deformed (planarity, symmetry) mainly because of the high degree of substitution. The two sodium ions, located within pockets of the anion rods, are coordinated by six and seven O atoms, resulting in octa­hedral and penta­gonal-bipyramidal coordinations, respectively. In addition to these coordinative bonds towards sodium, an extended network of intra- and inter­molecular hydrogen bonds occurs.

## Related literature

For synthetic procedures for 3,4,5-trihy­droxy­benzene­sulfonic acid, see: Pješčić *et al.* (2000 ▶). For the properties and application of cunitic (*i.e.* wedge-shaped, amphiphilic) gelator molecules,, see: Beginn *et al.* (2008 ▶); Zhu *et al.* (2004 ▶, 2006 ▶); Percec *et al.* (2004 ▶, 2006 ▶). For oxidation processes of pyrogallol, see: Siegel & Siegel (1950 ▶).
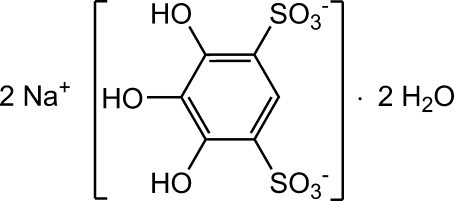


## Experimental

### 

#### Crystal data

2Na^+^·C_6_H_4_O_9_S_2_^2−^·2H_2_O
*M* *_r_* = 366.22
Triclinic, 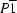

*a* = 6.9282 (4) Å
*b* = 9.1952 (5) Å
*c* = 10.6171 (6) Å
α = 68.303 (3)°
β = 75.984 (3)°
γ = 68.455 (2)°
*V* = 580.10 (6) Å^3^
*Z* = 2
Mo *K*α radiation
μ = 0.60 mm^−1^
*T* = 100 K
0.31 × 0.08 × 0.06 mm

#### Data collection

Bruker APEXII diffractometer
Absorption correction: multi-scan (*SADABS*; Bruker, 2008 ▶) *T*_min_ = 0.839, *T*_max_ = 0.965
24130 measured reflections
2000 independent reflections
1735 reflections with *I* > 2σ(*I*)
*R*_int_ = 0.041

#### Refinement

*R*[*F* ^2^ > 2σ(*F* ^2^)] = 0.026
*wR*(*F* ^2^) = 0.065
*S* = 1.09
2000 reflections
192 parameters
H-atom parameters constrained
Δρ_max_ = 0.46 e Å^−3^
Δρ_min_ = −0.40 e Å^−3^

### 

Data collection: *APEX2* (Bruker, 2008 ▶); cell refinement: *SAINT* (Bruker, 2008 ▶); data reduction: *SAINT*; program(s) used to solve structure: *SHELXS97* (Sheldrick, 2008 ▶); program(s) used to refine structure: *SHELXL97* (Sheldrick, 2008 ▶); molecular graphics: *DIAMOND* (Brandenburg, 2008 ▶); software used to prepare material for publication: *SHELXTL* (Sheldrick, 2008 ▶).

## Supplementary Material

Crystal structure: contains datablocks I, global. DOI: 10.1107/S1600536810036160/im2221sup1.cif

Structure factors: contains datablocks I. DOI: 10.1107/S1600536810036160/im2221Isup2.hkl

Additional supplementary materials:  crystallographic information; 3D view; checkCIF report

## Figures and Tables

**Table 1:** Hydrogen-bond geometry (Å, °)

*D*—H⋯*A*	*D*—H	H⋯*A*	*D*⋯*A*	*D*—H⋯*A*
O4—H4⋯O33	0.80	2.28	2.975 (2)	146
O4—H4⋯O13^i^	0.80	2.28	2.882 (2)	133
O5—H5⋯O1^ii^	0.80	1.99	2.738 (2)	156
O6—H6⋯O13	0.80	1.94	2.686 (2)	154
O1—H11⋯O2^iii^	0.81	1.99	2.793 (2)	173
O1—H12⋯O31^iv^	0.81	2.50	3.171 (2)	141
O1—H12⋯O12^iii^	0.81	2.53	3.080 (2)	127
O2—H21⋯O12^v^	0.81	2.15	2.926 (2)	162
O2—H22⋯O31^v^	0.81	2.30	3.064 (2)	159

Symmetry codes: (i) 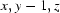; (ii) 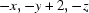; (iii) 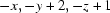; (iv) 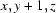; (v) 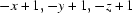.

**Fig. 1.**
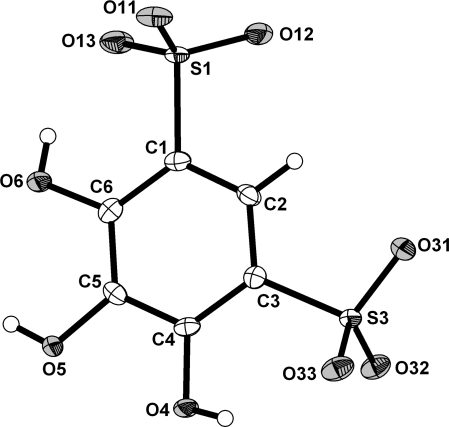
: Ball-and-stick model of the 4,5,6-trihydroxybenzene-1,3-disulfonate ion, with the atomic numbering scheme; with exception of the hydrogen atoms, which are shown as spheres with common isotropic radius, all other atoms are represented as thermal displacement ellipsoids showing 50% probability level of the corresponding atoms.

**Fig. 2.**
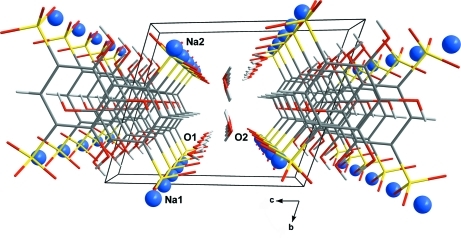
: Ball-and-stick model of the crystal packing structure of the title compound in direction of the a-axis with the atomic numbering scheme used for the sodium ions and the oxygen atoms of the water molecules; for clarity all atoms are shown as spheres with common isotropic radius.

**Fig. 3.**
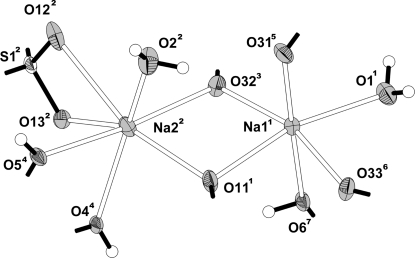
: Ball-and-stick representation of the sodium environment; with exception of the hydrogen atoms, which are shown as spheres with common isotropic radius, all other atoms are represented as thermal displacement ellipsoids showing 50% probability level of the corresponding atom; covalent bonds as black rods, coordinative bonds as white ones; symmetry codes: (1) x, y, z; (2) -1 + x, y, z; (3) -1 + x, 1 + y, z; (4) -x, 1 - y, z; (5) -x, 1 - y, 1 - z; (6) x, 1 + y, z; (7) -x, 2 - y, -z.

**Fig. 4.**
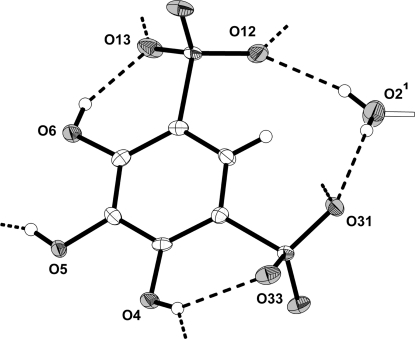
: Ball-and-stick model of the hydrogen-bond arrangement the 4,5,6-trihydroxybenzene-1,3-disulfonate ion is involved in; with exception of the hydrogen atoms, which are shown as spheres with common isotropic radius, all other atoms are represented as thermal displacement ellipsoids showing 50% probability level of the corresponding atoms; symmetry codes: (1) 1 - x,1 - y,1 - z.

## supplementary crystallographic information

### Comment

Cunitic compounds are amphiphilic substances consisting of wedge shaped molecules with the polarity of the wedges tip to be considerably different from the polarity of the wedges base (Beginn *et al.*, 2008). In bulk and in solution of non - semi polar solvents the wedges selfassemble in form of cylindrical superstructures with the polar tip arranged in the centre of the cylinder, while the non-polar base is directed to the cylinders outer surface. In bulk the cylinders arrange in regular patterns and frequently form columnar mesophases, while in diluted solutions (< 1 - 5 wt%) isolated cylinders as well as cylinder bundles tend to form three-dimensional networks that confine the solvent and cause the macroscopic gelation of the liquid (Percec *et al.*, 2006).

Based on this peculiar structure formation, numerous investigations have been devoted to the subject. However, since the formation of cylindrical superstructures is a common phenomenon for cunitic molecules and since it occurs almost independently of the actual chemistry, *i.e.* the functional groups making the molecular tip of the compounds, the cylinders can be exploited to generate molecular defined channels to transport molecules, ions, photons or electrons (Percec *et al.*, 2004).

Functional membranes containing self-organized supramolecular channels of cunitic molecules with crown-ether and carboxylate functional tips have been prepared. Presently there is ongoing research to generate supramolecular channels containing sulfonate groups, since these can be used as model materials to investigate the "superselectivity" effects of Nafion and other perfluoro – sulfonate membranes (Zhu *et al.*, 2006, 2004).

In research for new starting materials for cunitic compounds we tried to prepare 3,4,5-trihydroxybenzenesulfonic acid by sulfonation of 1,2,3-trihydroxybenzene. However, in applying the reaction conditions and work-up procedures described for this compound (Pješčić *et al.*, 2000) only one compound could be isolated in very low yield. Subsequent spectroscopic measurements of this compound gave evidence that the product corresponds more to a "disulfonate" than to the desired "monosulfonate". Finaly these observations were confirmed by a single-crystal X-ray structure determination, the results of which we present here.

From bond lengths [mean value: 1.391 (9) Å, range: 1.380 (3) – 1.399 (3) Å] and bond angles [mean value: 120.0 (7)°, range: 119.8 (2)° - 121.1 (2)°] the benzene ring of the 4,5,6-trihydroxybenzene-1,3-disulfonate ion looks very regular (Fig. 1) but a detailed conformation analysis including the directly bonded oxygen and sulfur atoms results in some significant deviations from planarity as well as from geometry of a regular hexagon. Deviations from planarity of the benzene ring itself can be best described by use of two least squares planes [deviations from plane 1: C1 = 0.004 (2) Å; C2 = -0.004 (3) Å; C3 = 0.002 (2) Å; C6 = -0.002 (2) Å; plane 2: C3 = 0.004 (2) Å; C4 = -0.008 (2) Å; C5 = 0.008 (2) Å; C6 = -0.004 (2) Å] showing a dihedral angle of 3.4 (2)°. Much greater deviations from planarity are found for the carbon bonded oxygen and sulfur atoms, which are far away from these two least-squares planes mainly as a result of their steric repulsion: O4 = -0.014 (2) from plane 2; O5 = 0.060 (2) from plane 2; O6 = -0.051 (2) from plane 1 and 0.043 (2) from plane 2; S1 = -0.012 (1) from plane 1; S3 = -0.190 (1) from plane 1 and 0.201 (1) from plane 2.

The deviations from the geometry of a regular hexagon of the benzene ring itself are small (with respect to bond lengths and angles) those taking the substituents into account are considerable: neither are adjacent bonds parallel to each other nor are these bonds directed to the centre of the benzene ring. On the other side, carbon-oxygen [1.361 (3) - 1.363 (2) Å, mean value 1.362 (1) Å] and carbon-sulfur [1.770 (2) Å for S1, 1.760 (2) Å for S3] bond lengths are in the range expected for single bonds between these atoms.

In the crystal structure, anions are stacked parallel [distances: 3.275 (1) Å and 3.411 (1) Å] to each other forming rods along the crystallographic *a* axis with a crystallographic centre of symmetry between each pair of anions. Therefore the anions within the rods change their orientation from one to another. Based on a mismatch of the anion centre from the *a* axis, the benzene rings are not completely congruent within these rods. Thus, possible π - π interactions between neighbouring benzene rings are restricted to only two carbon atoms (or one bond).

The sodium ions are located in pockets of the rods formed by the organic anions, whereas the two additional water molecules are situated between them (Fig. 2). In summary, the sodium ions are coordinated by 6 [Na1] and 7 [Na2] oxygen atoms of the water molecules, hydroxyl groups and SO_3_-groups resulting in an octahedral and pentagonal-bipyramidal coordination, respectively. The pentagonal-bipyramidal coordination is caused by a SO_3_-group coordinating as a bidental ligand to one of the sodium cations [Na2]. Both coordination polyhedrons are linked to each other *via* a common edge built up by two oxygen atoms of two different SO_3_-groups (Fig. 3).

Besides the already described interactions, there is an extended network of intra- and intermolecular hydrogen bonds. Those in which the 4,5,6-trihydroxybenzene-1,3-disulfonate ion is involved in are shown in Fig. 4.

### Experimental

*Synthesis:*

Pyrogallol (1,2,3-trihydroxybenzene 1) was sulfonated according to (Pješčić *et al.*, 2000) by mixing 3.78 g (29.97 mmol) 1,2,3-trihydroxybenzene and 100 ml of concentrated sulfuric acid in a 250 ml round bottomed flask at 25 °C with stirring. Within 30 minutes the educt dissolved and the reaction mixture was stirred for another 48 h at 25°C. Subsequently the white precipitate was filtered over a P4 glass frit and the solid residue was redissolved in 20 ml water. After the solution was allowed to cool to ambient temperature it was rapidly mixed with a solution of 6 g sodium chloride in 17 ml water. After storing at 5°C over night in a refrigerator, the precipitated white crystals were collected by filtration over a P4 glass frit. In contrast to literature reports the crude product was hardly soluble in ethanol, but could be recrystallized twice from 10 ml of water to remove inorganic sodium salts. The final product 2 was dried in a desiccator at ambient temperature *in vacuo* over phosphorpentoxide until weight constancy. Yield: 1.15 g = 11.6% of theory, white crystals. After purification, the crystals changed their colour from white to a light red, which is caused by oxidation processes being typical for pyrogallol (Siegel & Siegel, 1950).

33.2 ± 0.1 mg of compound 2 were heated in air at 700 °C for 24 h to yield 14.2 ± 0.1 mg white ash. A complete combustion to sodium sulfate should theoretically yield a residual mass of 14.277 mg hence compound 2 was virtually free of sodium chloride. This finding was supported by an elemental analysis stating a chloride content of only 0.39%. Elemental analysis ('Mikroanalytisches Laboratorium H. Kolbe', Mülheim a.d. Ruhr, Germany): Na_2_[C_6_H(OH)_3_(SO_3_)_2_], C: 21.71(21.82), H: 1.19 (1.22), S 19.12 (19.42).

*Spectroscopic studies:*

^1^H–, and ^13^C-NMR spectra were measured in D_2_O containing TMS as internal standard on a Bruker DPX-250 F T-NMR Spectrometer at 250, and 62.5 MHz, respectively. Chemical shifts refer to the solvent signal at 4.79 p.p.m. (^1^H-NMR, HDO) and 39.43 p.p.m (^13^C-NMR, DMSO). ^1^H-NMR (D_2_O, δ/p.p.m.): 7.622 S, ^13^C-NMR (D_2_O, δ/p.p.m): 146.7, 133.4, 120.8, 118.7. The ^1^H-NMR spectrum was measured from a mixture of 11.9 mg 2 and 8.9 mg (0.101 mmol) 1,4-dioxane. The integrated signal intensity of 2 (δ = 7.62 p.p.m) to dioxane (δ = 3.74 p.p.m) of 1: 24(±1) was incompatible with a monosulfonated product, Na[C_6_H_2_(OH)_3_(SO_3_)] [expected signal intensity ratio = 1: 7.7] but fitted well to a disulfonated compound, Na_2_[C_6_H(OH)_3_(SO_3_)_2_] [expected signal intensity ratio = 1: 22.4].

Attenuated Total Reflection Infrared spectra were obtained with a Bruker Vertex 70 F T—IR photospectrometer equipped with Golden-Gate-Diamond-ATR reflection device. The substances were pressed on the ATR crystal and measured in reflection. IR (ν/cm^-1^): 3162 (broad), 1620.0, 1496.6, 1465.7, 1336.5, 1222.7, 1159.1, 1072.3, 1049.2, 1024.1, 883.3, 790.7, 748.3, 605.6.

*Crystallographic studies:*

For single-crystal X-Ray analysis a suitable single-crystal was selected under a polarization microscope and mounted on a 400/25 µm MicroMesh MiTeGen^TM^ using FOMBLIN Y perfluropolyether (LVAC 16/6, Aldrich).

### Refinement

Hydrogen atoms were clearly identified in difference Fourier syntheses. The positions of hydrogen atoms bonded to oxygen were refined with respect to two common O–H bond lengths (H_2_O: 0.81 Å; –OH: 0.80 Å), and an idealized H–O–H angle of 104.5°, before they were allowed to ride on the corresponding oxygen atoms. The hydrogen atom bonded to the carbon atom of the benzene ring was refined at a calculated position riding on the carbon atom with C–H = 0.95 Å. Two equivalent displacement factors were refined for hydrogen atoms: one for the four hydrogen atoms of the organic molecule and one for the four hydrogen atoms of the two water molecules. All other atoms were refined with anisotropic displacement parameters.

### Crystal data

**Table:**

2Na^+^·C_6_H_4_O_9_S_2_^2^^−^·2H_2_O	*Z* = 2
*M**_r_* = 366.22	*F*(000) = 372
Triclinic, *P*1	*D*_x_ = 2.097 Mg m^−^^3^
Hall symbol: -P 1	Mo *K*α radiation, λ = 0.71073 Å
*a* = 6.9282 (4) Å	Cell parameters from 9296 reflections
*b* = 9.1952 (5) Å	θ = 2.7–27.8°
*c* = 10.6171 (6) Å	µ = 0.60 mm^−^^1^
α = 68.303 (3)°	*T* = 100 K
β = 75.984 (3)°	Needle, red
γ = 68.455 (2)°	0.31 × 0.08 × 0.06 mm
*V* = 580.10 (6) Å^3^	

### Data collection

**Table:**

Bruker APEXII diffractometer	2000 independent reflections
Radiation source: fine-focus sealed tube	1735 reflections with *I* > 2σ(*I*)
graphite	*R*_int_ = 0.041
phi and ω scans	θ_max_ = 25.0°, θ_min_ = 2.1°
Absorption correction: multi-scan (*SADABS*; Bruker, 2008)	*h* = −8→8
*T*_min_ = 0.839, *T*_max_ = 0.965	*k* = −10→10
24130 measured reflections	*l* = −12→12

### Refinement

**Table:**

Refinement on *F*^2^	Primary atom site location: structure-invariant direct methods
Least-squares matrix: full	Secondary atom site location: difference Fourier map
*R*[*F*^2^ > 2σ(*F*^2^)] = 0.026	Hydrogen site location: inferred from neighbouring sites
*wR*(*F*^2^) = 0.065	H-atom parameters constrained
*S* = 1.09	*w* = 1/[σ^2^(*F*_o_^2^) + (0.0278*P*)^2^ + 0.5372*P*] where *P* = (*F*_o_^2^ + 2*F*_c_^2^)/3
2000 reflections	(Δ/σ)_max_ < 0.001
192 parameters	Δρ_max_ = 0.46 e Å^−^^3^
0 restraints	Δρ_min_ = −0.40 e Å^−^^3^

### Special details

**Table:**

Geometry. All e.s.d.'s (except the e.s.d. in the dihedral angle between two l.s. planes) are estimated using the full covariance matrix. The cell e.s.d.'s are taken into account individually in the estimation of e.s.d.'s in distances, angles and torsion angles; correlations between e.s.d.'s in cell parameters are only used when they are defined by crystal symmetry. An approximate (isotropic) treatment of cell e.s.d.'s is used for estimating e.s.d.'s involving l.s. planes.
Refinement. Refinement of *F*^2^ against ALL reflections. The weighted *R*-factor *wR* and goodness of fit *S* are based on *F*^2^, conventional *R*-factors *R* are based on *F*, with *F* set to zero for negative *F*^2^. The threshold expression of *F*^2^ > σ(*F*^2^) is used only for calculating *R*-factors(gt) *etc*. and is not relevant to the choice of reflections for refinement. *R*-factors based on *F*^2^ are statistically about twice as large as those based on *F*, and *R*- factors based on ALL data will be even larger.

### Fractional atomic coordinates and isotropic or equivalent isotropic displacement parameters (Å^2^)

**Table:**

	*x*	*y*	*z*	*U*_iso_*/*U*_eq_	
C1	0.1954 (3)	0.5924 (3)	0.1388 (2)	0.0094 (5)	
C2	0.1960 (3)	0.4422 (3)	0.2347 (2)	0.0103 (5)	
H2	0.1730	0.4321	0.3291	0.025 (4)*	
C3	0.2296 (3)	0.3064 (3)	0.1960 (2)	0.0087 (4)	
C4	0.2534 (3)	0.3218 (3)	0.0577 (2)	0.0085 (4)	
C5	0.2577 (3)	0.4724 (3)	−0.0403 (2)	0.0093 (5)	
C6	0.2323 (3)	0.6076 (3)	−0.0004 (2)	0.0092 (5)	
O4	0.2764 (2)	0.19739 (17)	0.00969 (14)	0.0109 (3)	
H4	0.2447	0.1257	0.0723	0.025 (4)*	
O5	0.2915 (2)	0.47596 (18)	−0.17322 (14)	0.0114 (3)	
H5	0.2801	0.5660	−0.2260	0.025 (4)*	
O6	0.2426 (2)	0.75022 (17)	−0.10051 (14)	0.0120 (3)	
H6	0.2476	0.8099	−0.0635	0.025 (4)*	
S1	0.15178 (8)	0.75784 (6)	0.20027 (5)	0.00896 (14)	
O11	−0.0728 (2)	0.83560 (18)	0.21945 (15)	0.0144 (3)	
O12	0.2449 (3)	0.68521 (18)	0.32711 (16)	0.0173 (4)	
O13	0.2611 (2)	0.86540 (18)	0.09335 (15)	0.0163 (4)	
S3	0.25656 (8)	0.11379 (6)	0.32262 (5)	0.00844 (14)	
O31	0.1976 (2)	0.14814 (18)	0.45258 (15)	0.0148 (4)	
O32	0.4746 (2)	0.01570 (18)	0.30296 (15)	0.0124 (3)	
O33	0.1180 (2)	0.04470 (18)	0.29754 (15)	0.0140 (4)	
Na1	−0.21766 (13)	1.04339 (10)	0.32841 (8)	0.0108 (2)	
Na2	0.59706 (14)	0.77666 (10)	0.22482 (8)	0.0142 (2)	
O1	−0.2793 (3)	1.26461 (18)	0.40886 (15)	0.0164 (4)	
H11	−0.3886	1.2910	0.4554	0.045 (5)*	
H12	−0.1912	1.2450	0.4555	0.045 (5)*	
O2	0.6746 (3)	0.62770 (19)	0.45172 (16)	0.0196 (4)	
H21	0.7198	0.5353	0.5011	0.045 (5)*	
H22	0.7376	0.6789	0.4635	0.045 (5)*	

### Atomic displacement parameters (Å^2^)

**Table:**

	*U*^11^	*U*^22^	*U*^33^	*U*^12^	*U*^13^	*U*^23^
C1	0.0076 (11)	0.0091 (11)	0.0130 (11)	−0.0014 (9)	−0.0017 (9)	−0.0059 (9)
C2	0.0107 (11)	0.0120 (11)	0.0087 (11)	−0.0023 (10)	−0.0006 (9)	−0.0054 (9)
C3	0.0064 (11)	0.0092 (11)	0.0098 (10)	−0.0016 (9)	−0.0010 (8)	−0.0030 (9)
C4	0.0051 (10)	0.0084 (11)	0.0133 (11)	−0.0009 (9)	−0.0011 (8)	−0.0059 (9)
C5	0.0069 (11)	0.0132 (11)	0.0087 (11)	−0.0024 (9)	−0.0008 (8)	−0.0053 (9)
C6	0.0042 (10)	0.0092 (11)	0.0127 (11)	−0.0011 (9)	−0.0011 (8)	−0.0028 (9)
O4	0.0170 (8)	0.0074 (7)	0.0102 (7)	−0.0061 (7)	0.0003 (6)	−0.0037 (6)
O5	0.0191 (8)	0.0080 (8)	0.0070 (7)	−0.0044 (7)	−0.0014 (6)	−0.0021 (6)
O6	0.0189 (9)	0.0084 (8)	0.0106 (8)	−0.0059 (7)	−0.0016 (6)	−0.0035 (6)
S1	0.0108 (3)	0.0075 (3)	0.0104 (3)	−0.0026 (2)	−0.0010 (2)	−0.0052 (2)
O11	0.0111 (8)	0.0151 (8)	0.0218 (9)	−0.0033 (7)	−0.0005 (6)	−0.0129 (7)
O12	0.0253 (9)	0.0109 (8)	0.0181 (8)	−0.0005 (7)	−0.0111 (7)	−0.0071 (7)
O13	0.0224 (9)	0.0138 (8)	0.0161 (8)	−0.0104 (7)	0.0049 (7)	−0.0081 (7)
S3	0.0103 (3)	0.0072 (3)	0.0087 (3)	−0.0028 (2)	−0.0011 (2)	−0.0033 (2)
O31	0.0238 (9)	0.0114 (8)	0.0091 (8)	−0.0051 (7)	−0.0012 (7)	−0.0042 (6)
O32	0.0091 (8)	0.0099 (8)	0.0182 (8)	−0.0014 (7)	−0.0028 (6)	−0.0053 (6)
O33	0.0125 (8)	0.0109 (8)	0.0212 (9)	−0.0044 (7)	−0.0026 (7)	−0.0066 (7)
Na1	0.0119 (4)	0.0103 (4)	0.0115 (4)	−0.0041 (4)	−0.0011 (3)	−0.0045 (3)
Na2	0.0184 (5)	0.0132 (4)	0.0135 (4)	−0.0051 (4)	0.0000 (4)	−0.0079 (4)
O1	0.0215 (9)	0.0143 (8)	0.0144 (8)	−0.0044 (7)	−0.0027 (7)	−0.0062 (7)
O2	0.0266 (10)	0.0156 (9)	0.0191 (9)	−0.0084 (8)	−0.0093 (7)	−0.0025 (7)

### Geometric parameters (Å, °)

**Table:**

C1—C2	1.380 (3)	S1—O11	1.4530 (16)
C2—C3	1.379 (3)	S1—O12	1.4545 (16)
C3—C4	1.396 (3)	S1—O13	1.4644 (15)
C4—C5	1.397 (3)	O11—Na1	2.3791 (17)
C5—C6	1.396 (3)	O12—Na2	2.7178 (18)
C6—C1	1.399 (3)	O13—Na2	2.7143 (18)
C1—S1	1.770 (2)	S3—O33	1.4465 (15)
C2—H2	0.9500	S3—O31	1.4577 (16)
C3—S3	1.760 (2)	S3—O32	1.4579 (15)
C4—O4	1.361 (3)	Na1—O1	2.3495 (17)
C5—O5	1.363 (2)	Na2—O2	2.3695 (17)
C6—O6	1.363 (2)	O1—H11	0.8056
O4—H4	0.7979	O1—H12	0.8056
O5—H5	0.7979	O2—H21	0.8056
O6—H6	0.7980	O2—H22	0.8056
			
C2—C1—C6	119.77 (19)	C1—S1—Na2	109.02 (7)
C2—C1—S1	117.18 (16)	S1—O11—Na1	120.84 (8)
C6—C1—S1	123.04 (16)	S1—O12—Na2	96.74 (8)
C3—C2—C1	121.12 (19)	S1—O13—Na2	96.63 (8)
C3—C2—H2	119.4	O33—S3—O31	113.52 (9)
C1—C2—H2	119.4	O33—S3—O32	111.33 (9)
C4—C3—C2	119.84 (19)	O31—S3—O32	113.24 (9)
C2—C3—S3	118.86 (16)	O33—S3—C3	106.02 (9)
C4—C3—S3	121.20 (16)	O31—S3—C3	105.58 (10)
O4—C4—C3	124.23 (18)	O32—S3—C3	106.43 (9)
O4—C4—C5	116.35 (18)	O1—Na1—O11	165.05 (6)
C5—C4—C3	119.41 (19)	O1—Na1—S1	144.75 (5)
O5—C5—C6	123.60 (18)	O11—Na1—S1	21.77 (4)
O5—C5—C4	116.09 (19)	O2—Na2—O13	134.47 (6)
C6—C5—C4	120.30 (19)	O2—Na2—O12	81.64 (5)
O6—C6—C5	117.69 (18)	O13—Na2—O12	53.02 (5)
O6—C6—C1	122.94 (19)	O2—Na2—S1	108.18 (5)
C1—C6—C5	119.37 (19)	O13—Na2—S1	26.77 (3)
C4—O4—H4	107.7	O12—Na2—S1	26.57 (3)
C5—O5—H5	113.1	O2—Na2—H22	17.3
C6—O6—H6	106.5	O13—Na2—H22	146.5
O11—S1—O12	113.02 (9)	O12—Na2—H22	96.0
O11—S1—O13	112.95 (9)	S1—Na2—H22	122.3
O12—S1—O13	112.34 (9)	Na1—O1—H11	117.6
O11—S1—C1	107.38 (9)	Na1—O1—H12	110.7
O12—S1—C1	105.84 (9)	H11—O1—H12	104.6
O13—S1—C1	104.55 (9)	Na2—O2—H21	141.7
O11—S1—Na2	143.60 (6)	Na2—O2—H22	101.8
O12—S1—Na2	56.69 (7)	H21—O2—H22	104.6
O13—S1—Na2	56.60 (7)		

### Hydrogen-bond geometry (Å, °)

**Table:**

*D*—H···*A*	*D*—H	H···*A*	*D*···*A*	*D*—H···*A*
O4—H4···O33	0.80	2.28	2.975 (2)	146
O4—H4···O13^i^	0.80	2.28	2.882 (2)	133
O5—H5···O1^ii^	0.80	1.99	2.738 (2)	156
O6—H6···O13	0.80	1.94	2.686 (2)	154
O1—H11···O2^iii^	0.81	1.99	2.793 (2)	173
O1—H12···O31^iv^	0.81	2.50	3.171 (2)	141
O1—H12···O12^iii^	0.81	2.53	3.080 (2)	127
O2—H21···O12^v^	0.81	2.15	2.926 (2)	162
O2—H22···O31^v^	0.81	2.30	3.064 (2)	159

Symmetry codes: (i) *x*, *y*−1, *z*; (ii) −*x*, −*y*+2, −*z*; (iii) −*x*, −*y*+2, −*z*+1; (iv) *x*, *y*+1, *z*; (v) −*x*+1, −*y*+1, −*z*+1.
